# Day to day work as a psychiatrist in the psycho-oncology department

**DOI:** 10.1192/j.eurpsy.2025.1782

**Published:** 2025-08-26

**Authors:** J. Knific, A. C. Skufca Smdrel, S. Kocijančič Azzaoui

**Affiliations:** 1 Psycho-oncology, Institute of Oncology, ljubljana, Slovenia

## Abstract

**Introduction:**

Liaison psychiatry mostly deals with consultations that are required in different department of somatic hospitals. The patients range from surgery to dermatology and the consultations vary depending on the department. As far as we know, in Europe, there is no psycho-oncology department with a psychiatrist on board full-time. In Ljubljana, Slovenia, this has been the case since 1984, with a short break for a few years, but an addition again in the last three years. Working as a psychiatrist in a very specific and demanding environment at oncology has its; challenges, as well as integrating the work with a team of clinical psychologists.

**Objectives:**

We wanted to analyse how many and what kind of patients are referred to the psycho-oncology department, either to a psychiatrist or a clinical psychologist.

**Methods:**

We gathered the data from referrals; How many patients were checked in an office or in a hospital setting, how many of these were first referrals. We also compared the checkups by a psychiatrist or by a clinical psychologist. Out of these, we looked at the oncological diagnosis and if they were referred by an oncologist. Out of our first referrals checked which diagnosis was most prevalent.

**Results:**

In the year 2023 the statistical data of the psycho-oncology department was gathered by the psychiatrist and clinical psychologist. Number of referrals for a psychiatrist was 895 and for a clinical psychologist was 1511. The psychiatrist performed 215 first referrals, so 24% out of all checkups. The psychologist did 325 first referrals, which is 21% out of all check-ups. For the psychologist and the psychiatrist the most common adjoining cancer diagnosis with a referral was breast cancer, making up 47% in the psychiatrist case and 44% in the psychologist case of all of the first checkups. Most common indication for a referral to a psychologist was mental distress, while for a psychiatrist was depression. Interestingly both the psychologist and psychiatrist would afterwards diagnose their patients most often with the diagnosis of adjustment disorder.

**Conclusions:**

The data showed interesting differences and similarities between referrals to a psychologist and a psychiatrist. It is important to note, that the distinction of referrals to psychiatrist or clinical psychologist at the psychooncology department is rather new. It should be mentioned, that the distinction is also that there is only one psychiatrist compared to three psychologists. The data will continue to be gathered as to ascertain the adaptation of the entire oncology clinic to a better accessibility of a consultation psychiatrist.

**Disclosure of Interest:**

None Declared

